# Diagnosis and Management of Gallstone Ileus: An Individualized Approach Based on Clinical Presentation and Patient Comorbidities

**DOI:** 10.7759/cureus.87215

**Published:** 2025-07-03

**Authors:** Carlos Iskyam Zaldo Arredondo, Mayra Guzmán Ortiz, Mariana Castañeda Llanos, Jose Oswaldo Ferre Bello, Salvador Pelayo González

**Affiliations:** 1 General Surgery, Hospital General de León, León, MEX

**Keywords:** cholecystocholedochal fistula, cholelithiasis, gallstone ileum, mirizzi's syndrome, small-bowel obstruction

## Abstract

Gallstone ileus is an uncommon cause of mechanical intestinal obstruction. It results from the impaction of one or more gallstones within the gastrointestinal tract, usually after migrating through a cholecysto-duodenal fistula. The clinical presentation is nonspecific, including abdominal pain, vomiting, distension, and constipation. Computed tomography (CT) scan is the diagnostic modality of choice due to its high sensitivity. The treatment is surgical, with enterolithotomy being the initial procedure to extract the impacted stone.

This case report describes a 74-year-old woman diagnosed with gallstone ileus, who was managed with a simple enterolithotomy without repair of the fistula or cholecystectomy, given her high surgical risk. Primary management of gallstone ileus should focus on resolving the intestinal obstruction via enterolithotomy. Although performing cholecystectomy and fistula repair reduces the risk of recurrence, the decision must weigh the benefits against the risks associated with more complex surgical interventions in frail patients.

## Introduction

Intestinal obstruction is a mechanical interruption of intestinal transit that can be intrinsic or extrinsic to the lumen [1,2]. It is a common cause of hospital admission for acute abdominal pain. In the small intestine, the main causes are intestinal adhesions (55%-75%), hernias (15%-25%), and neoplasms (5%-10%) [1,2]. In the colon, cancer accounts for approximately 60% of cases, while volvulus and diverticular disease constitute the remaining 30% [2,3]. Other less frequent causes include endometriosis, foreign bodies, and bezoars, among which gallstone ileus is noteworthy [2-7].

Gallstone ileus is a rare complication of cholelithiasis that causes mechanical obstruction of the gastrointestinal tract due to impaction of one or more stones. It occurs when a gallstone migrates from the gallbladder or biliary ducts into the intestinal lumen through a fistula, with cholecysto-duodenal fistula being the most common [8]. This entity represents 1%-3% of all cases of intestinal obstruction, although its incidence can increase in patients over 65 years of age [5].

Symptoms of gallstone ileus are nonspecific and include spasmodic abdominal pain, nausea, vomiting, distension, and constipation [6,8,9]. An intermittent or subacute presentation may occur due to the gradual movement of the stone through the gastrointestinal tract until impaction [8].

Laboratory findings can include leukocytosis, elevated C-reactive protein, and electrolyte imbalances due to vomiting and dehydration associated with intestinal obstruction [1,8]. Liver function tests may be abnormal, showing hyperbilirubinemia, elevated transaminases, alkaline phosphatase, and gamma-glutamyl transpeptidase [6,8-11]. Lactate levels may be elevated, suggesting intestinal ischemia, although normal values do not rule out this complication [1].

For diagnosis, contrast-enhanced abdominal computed tomography (CT) is the modality of choice, with sensitivity exceeding 90% for confirming obstruction, identifying its cause, and assessing possible complications [1,2,5,8]. Although plain abdominal radiography and ultrasound can be used initially, their diagnostic value is limited compared to CT [2,6,8]. Recognition of ischemia signs, such as lack of bowel wall enhancement, mesenteric edema, or the closed-loop sign, is crucial, as these indicate the need for urgent surgical intervention [1,3].

An important radiologic finding in gallstone ileus diagnosis is the Rigler triad. It consists of the presence of radiopaque stones, pneumobilia (air within the biliary tree), and intestinal distension. Although considered pathognomonic, it appears in fewer than 50% of cases, so its absence does not exclude the diagnosis [5,7,8].

Management of gallstone ileus is predominantly surgical. Enterolithotomy, which involves removing the stone through an incision in the intestine, is the treatment of choice [4-8]. Preoperative stabilization of the patient, including fluid and electrolyte repletion, is essential before surgery [5,8].

## Case presentation

We received a 74-year-old female patient in the emergency department who was admitted with generalized abdominal distension, pain, vomiting, inability to tolerate oral intake, and absence of bowel movements for the past three days.

Her medical history included systemic arterial hypertension and chronic kidney disease without renal replacement therapy. She denied previous surgical procedures, diagnosed hernias, or a history of cholelithiasis. The patient had grade II obesity, with a body mass index (BMI) of 38.4.

Laboratory results at the time of admission are summarized in Table 1. 

**Table 1:** Laboratory results upon hospital admission.

Test	Patient result	Normal range
Glucose	170 mg/dL	74-106 mg/dL
Urea	133 mg/dL	15-36 mg/dL
Creatinine	5.4 mg/dL	0.52-1.04 mg/dL
Aspartate aminotransferase (AST)	30 U/L	4-35 U/L
Alanine aminotransferase (ALT)	32 U/L	14-36 U/L
Total bilirubin	1.2 mg/dL	0.2-1.3 mg/dL
Sodium	134 mmol/L	137-145 mmol/L
Potassium	4.3 mmol/L	3.5-5.1 mmol/L
Magnesium	2.9 mg/dL	1.6-2.3 mg/dL
Chloride	90 mmol/L	98-107 mmol/L
C-reactive protein (CRP)	389 mg/L	<10 mg/L
Leukocytes	12.32 x 10³/µL	4.00-10.00 x 10³/µL
Hemoglobin	16.7 g/dL	12.00-16.00 g/dL
Platelets	361 x 10³/µL	130-400 x 10³/µL
International normalized ratio (INR)	1.17	0.51-1.51

On physical examination, the patient was diaphoretic, reported generalized abdominal pain, and had absent bowel sounds. No hernias or previous surgical scars were evident. Urgent surgical intervention was not indicated at this time. A nasogastric tube was placed, draining an immediate output of 400 mL of bilious fluid.

The patient showed significant improvement following gastric decompression; however, symptoms recurred when attempting to resume oral intake. Consequently, a contrast-enhanced abdominal CT scan was performed.

**Figure 1:**
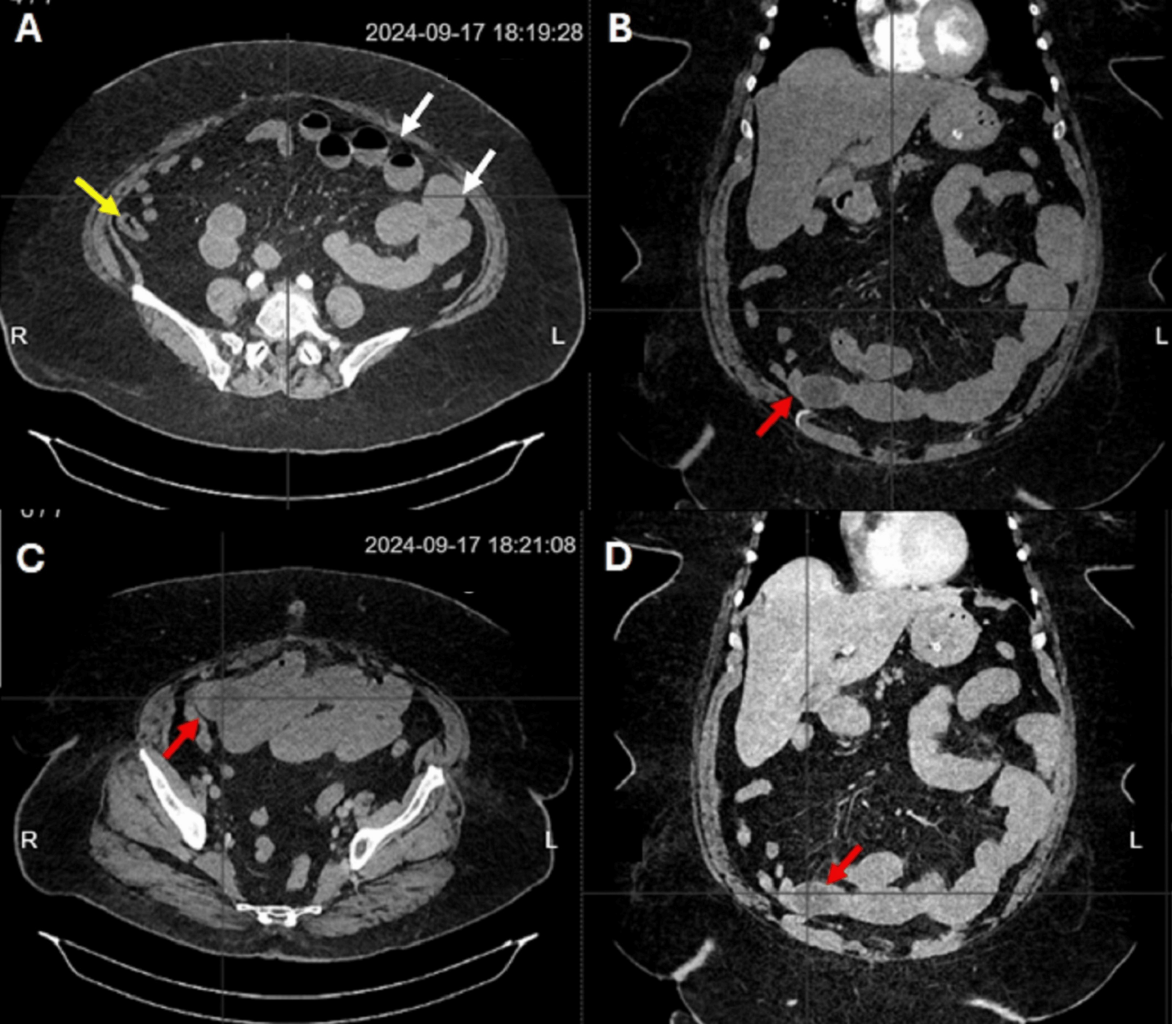
Contrast-enhanced abdominal CT showing signs compatible with distal intestinal obstruction. (A) Dilated small bowel loops with air-fluid levels (white arrow). The colon appears collapsed (yellow arrow), suggesting small bowel obstruction. (B, C, and D) Presence of a foreign body in the distal ileum causing a transitional zone (red arrow).

The CT revealed dilated bowel loops, air-fluid levels, and a collapsed colon. A foreign body was observed in the distal ileum, causing a transitional zone in the small intestine (Figure 1). Due to intestinal obstruction secondary to a foreign body, surgery was decided.

**Figure 2:**
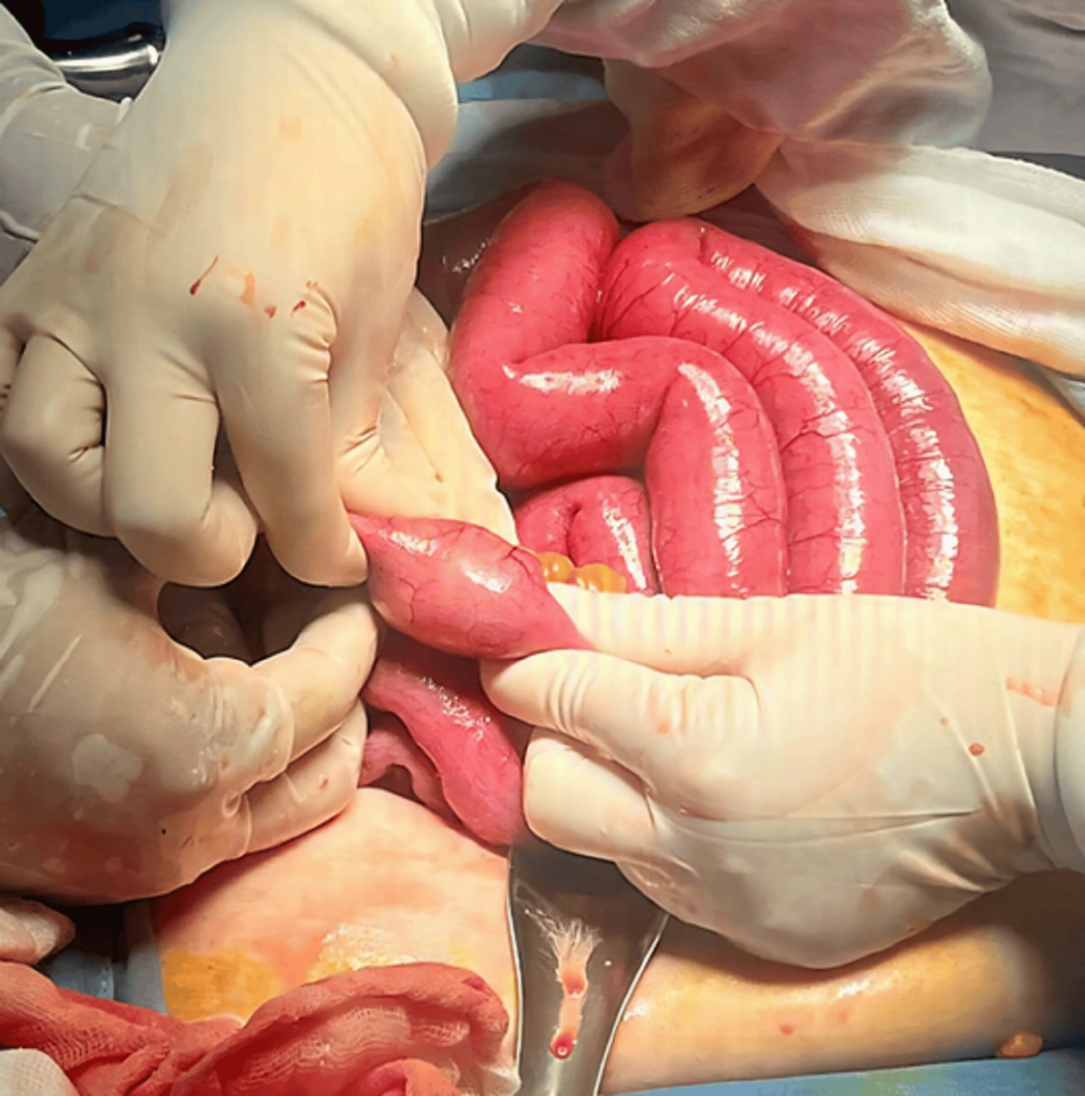
Foreign body in the distal ileum. Intraoperative image showing distended small bowel loops with evidence of an intraluminal foreign body located in the distal ileum.

**Figure 3:**
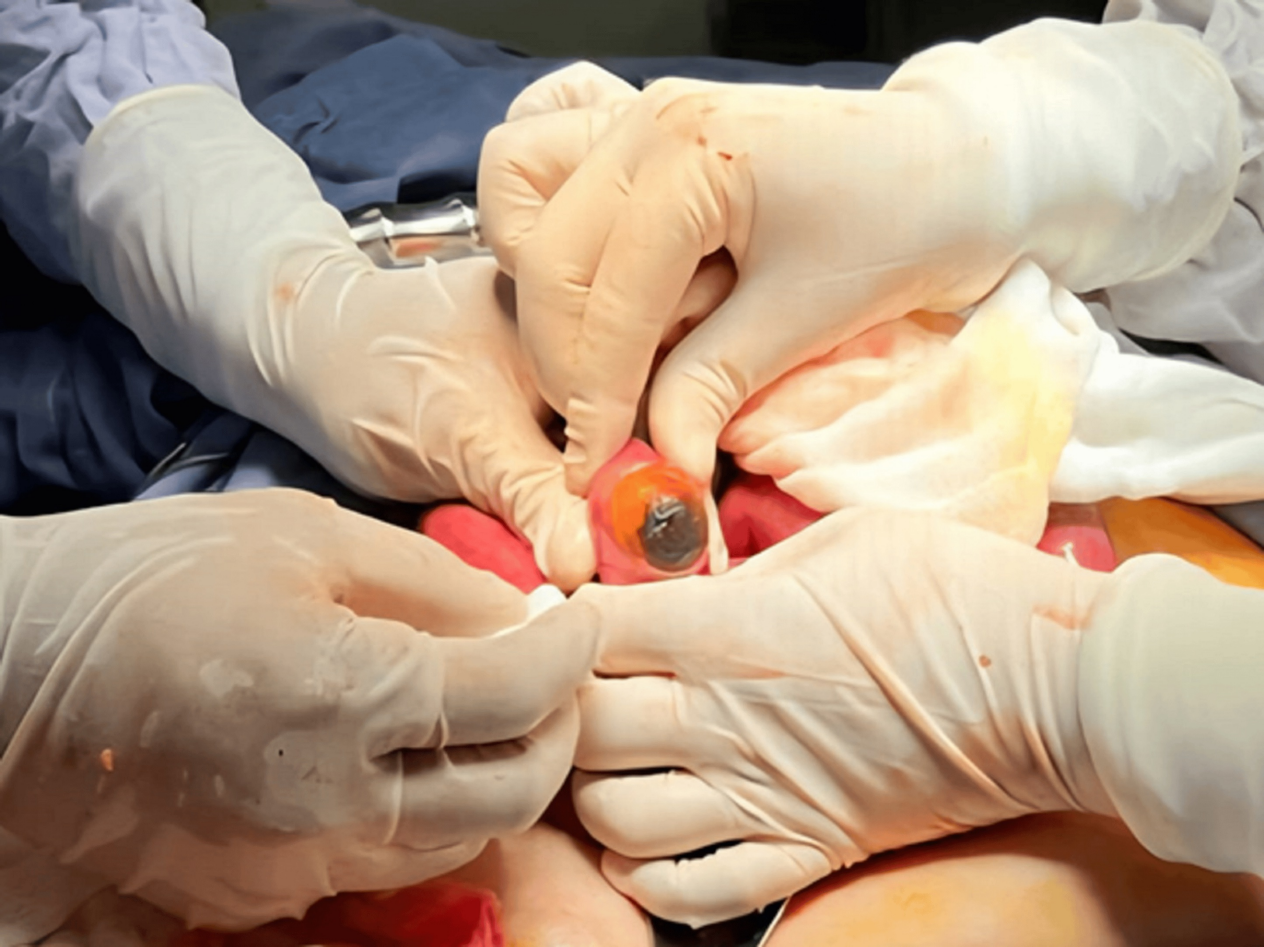
Enterolithotomy. Extraction of a gallstone causing intestinal obstruction, confirming gallstone ileus as the underlying cause of the clinical presentation.

**Figure 4:**
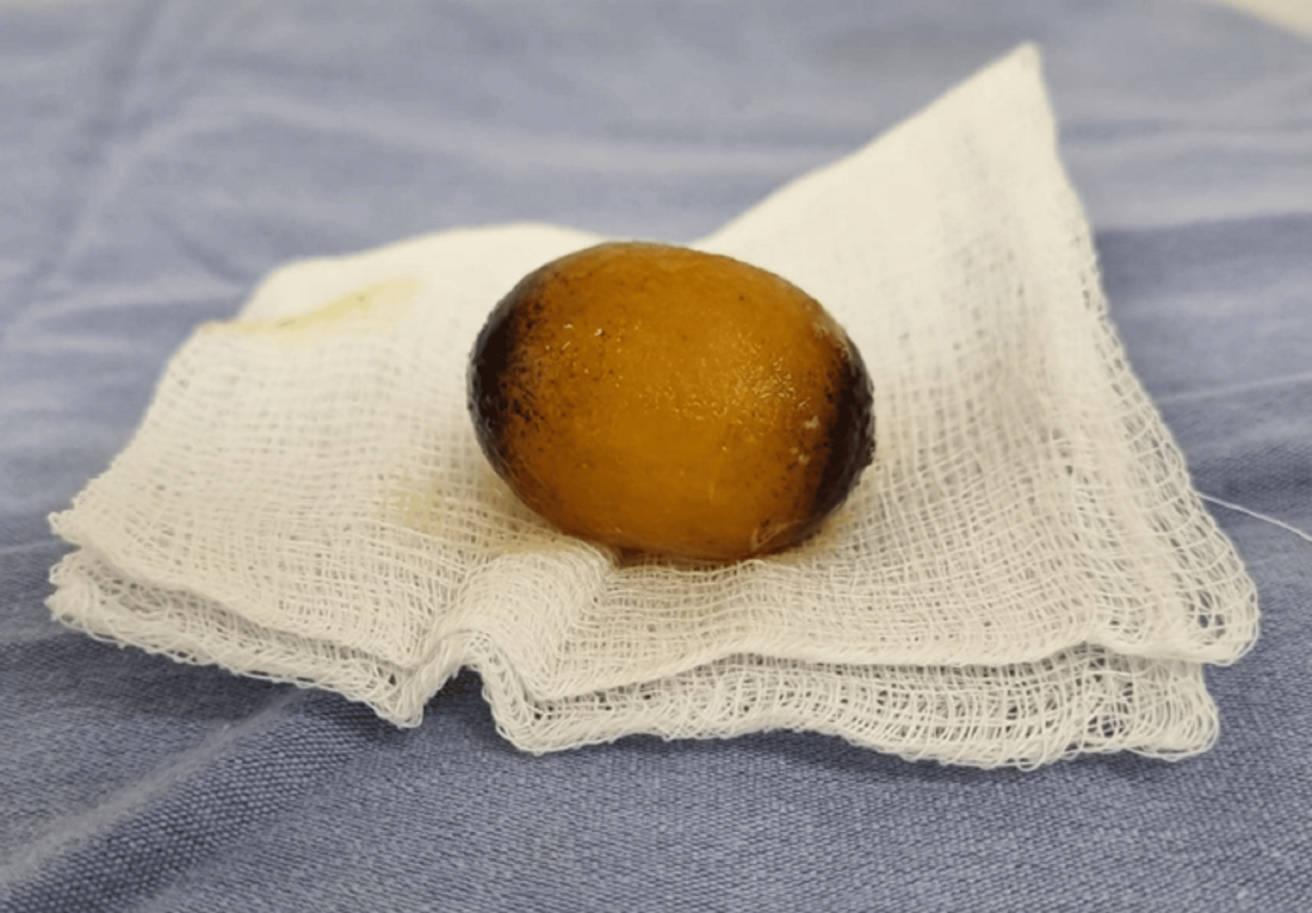
Giant gallstone. Macroscopic photograph of the extracted stone. The stone is oval-shaped, with a yellowish surface and pigmented edges, measuring 4 × 5 cm.

A laparotomy was performed, revealing dilated intestinal loops proximal to the foreign body located 40 cm from the ileocecal valve, which was obstructing the lumen (Figure 2). A simple enterolithotomy was carried out, removing a single stone measuring 5 x 4 cm (Figures 3, 4). The small intestine was sutured with a monofilament absorbable 3-0 suture in two layers. Given the risk factors presented by the patient, it was decided not to explore or repair the cholecysto-duodenal fistula, nor to perform a cholecystectomy; thus, the procedure was completed.

The patient had an uneventful recovery, being discharged on the sixth postoperative day. She tolerated an oral diet and reported normal bowel movements. No other interventions were necessary during her stay. During her last follow-up, she had not experienced any new episodes of intestinal obstruction or symptoms attributable to gallbladder pathology.

## Discussion

Gallstone ileus originates when a gallstone, typically larger than 2.5 cm in diameter, passes from the gallbladder into the intestinal lumen [5,8,12]. This occurs through an entero-biliary fistula, which is an abnormal communication between the gallbladder and the gastrointestinal tract [5,7-9,12]. The formation of this fistula usually results from recurrent episodes of cholecystitis, leading to inflammation, erosion, and necrosis of the gallbladder wall, facilitating the passage of stones into the intestinal lumen [5,6,8,12]. The most common fistula forms between the gallbladder and the duodenum due to their proximity (accounting for 85% of cases), although the stomach, jejunum, or colon can also be involved [8,12].

Surgical treatment is primary and can be performed via a laparoscopic approach, which offers lower morbidity; however, the conversion rate to open surgery is high (53.3%-93.3%) due to intestinal distension and severe inflammation [6-8,11,12].

The main surgical options include simple enterolithotomy, which involves removing the stone without repairing the biliary-enteric fistula or performing cholecystectomy [4,7,8,12]. A one-stage procedure that combines enterolithotomy, cholecystectomy, and fistula closure in a single operation, considered a more definitive approach that reduces long-term recurrence and biliary complications [4,8]; and a two-stage approach, involving an initial enterolithotomy followed by cholecystectomy and fistula repair in a subsequent procedure [5,8].

Recurrence of gallstone ileus has been reported between 2% and 8%, with a 10% risk of subsequent biliary complications, such as cholecystitis and cholangitis, and a potential up to 15% increased risk of gallbladder cancer in patients who do not undergo cholecystectomy or fistula repair [4,5,8,12]. However, performing cholecystectomy and fistula repair may not be necessary in all cases; treatment should be individualized based on the patient's condition [5,12].

Simple enterolithotomy is less complex, requires less operative time, and has a lower mortality rate of 11.7% compared to 16.9% with single-stage surgery [4,5,7,8,10,12]. Favorable outcomes have been reported with more conservative management, as some patients experience spontaneous fistula closure. Additionally, if the fistula persists, it can serve as an alternative drainage pathway, allowing for the natural expulsion of residual gallstones [5,7,8,10]. In this case, the decision to perform a simple enterolithotomy was made due to the patient's comorbidities. This resulted in an uneventful postoperative course with no recurrences to date.

## Conclusions

Gallstone ileus is a rare cause of intestinal obstruction requiring comprehensive management, including fluid-electrolyte resuscitation and stabilization, with contrast-enhanced CT as the diagnostic tool of choice. Surgical treatment primarily involves enterolithotomy to relieve the obstruction, with subsequent decisions on cholecystectomy and fistula repair based on patient stability and comorbidities. In high-risk or elderly patients, conservative management may be preferred, focusing on stone removal alone. Our case demonstrated successful resolution of obstruction through surgery without additional procedures, resulting in favorable short- and medium-term outcomes.
